# Innovative Management of Spastic Shoulder Contractures: A Retrospective Cohort Analysis of Combined Percutaneous Cryoneurolysis and Tenotomy

**DOI:** 10.3390/toxins18030137

**Published:** 2026-03-11

**Authors:** Paul Winston, Mahdis Hashemi, Fraser MacRae, Samuel Herzog, Maxime Billot, Romain David

**Affiliations:** 1 Vancouver Island Health Authority, Victoria, BC V8T 3P7, Canada; pauljwinston@gmail.com (P.W.); mahdishashemit@gmail.com (M.H.);; 2 Division of Physical Medicine and Rehabilitation, Faculty of Medicine, University of British Columbia, Vancouver, BC V5Z 2G9, Canada; 3 Canadian Advances in Neuro-Orthopedics for Spasticity Consortium (CANOSC), Kingston, ON K7K 1Z6, Canada; fraseramacrae@gmail.com; 4 School of Physical Therapy, Faculty of Health Sciences, Western University, London, ON N6A 3K7, Canada; 5 Department of Microbiology and Immunology, Faculty of Science, University of British Columbia, Vancouver, BC V6T 1Z4, Canada; 6 Centre Hospitalier Universitaire (CHU) de Poitiers, INSERM, Centre d’Investigation Clinique Domaine d’Innovation Technologique (CIC-IT) 1402, F-86000 Poitiers, France; maxime.billot@chu-poitiers.fr; 7 Université de Poitiers, CNRS, CeRCA, UMR 7295, F-86000 Poitiers, France; 8 Centre Hospitalier Universitaire (CHU) de Poitiers, Service de Médecine Physique et Réadaptation, F-86000 Poitiers, France

**Keywords:** spasticity, stroke, multiple sclerosis, cryoneurolysis, cerebral palsy, tenotomy, acquired brain injury

## Abstract

Shoulder spasticity is a common consequence of upper motor neuron lesions and may be associated with soft tissue contractures, limiting functional recovery. While both cryoneurolysis and tendon lengthening procedures are used individually in refractory cases, their combined effect has not been clearly established. It is consequently necessary to assess the efficacy of combining cryoneurolysis and percutaneous pectoral tenotomy in reducing shoulder spasticity and improving passive range of motion in patients with refractory shoulder spasticity and contracture. This retrospective, single-centre cohort study included 15 adults (≥19 years) with chronic shoulder spasticity and clinically confirmed musculotendinous contracture, previously treated with botulinum toxin injections without sufficient functional response, and free of pharmacological effects (last injection >4 months prior). All patients underwent cryoneurolysis targeting motor branches to the pectoral muscles. Outcomes included Modified Ashworth Scale (MAS) and shoulder Passive Range Of Motion (PROM). The combined approach provided significant improvements in spasticity severity for shoulder flexion (*p* < 0.01) and abduction (*p* < 0.01), and significant improvements in maximum PROM for shoulder flexion (*p* < 0.0001) and abduction (*p* < 0.0001). Combining cryoneurolysis and pectoral tenotomy appears feasible, safe, and clinically beneficial in selected patients with both spasticity and tendon contracture. Cryoneurolysis addresses the neural component, while tenotomy may restore mechanical excursion. This sequential diagnostic and therapeutic approach may enhance personalized management of mixed spastic–contracture shoulder limitations and could be applicable to other joints.

## 1. Introduction

Spasticity, defined as an abnormal increase in muscle tone, commonly occurs after central nervous system disorders such as Stroke, Multiple Sclerosis (MS), Spinal Cord Injury, Cerebral Palsy (CP) and Traumatic Brain Injury [1,2,3]. Approximately 20% to 40% of stroke survivors develop spasticity, significantly impacting health status, pain, function, and overall quality of life [4].

Among the multifaceted manifestations of upper limb spasticity, spastic shoulder contractures represent a particularly challenging clinical issue. These contractures, characterized by an increased muscle tone resulting in restricted shoulder movement, can lead to debilitating pain, hindered functional independence, and a diminished quality of life [5,6]. Whereas contractures most commonly develop as a consequence of severe and prolonged spasticity, the contribution of each component to the reduced range of motion and pain remains unclear. Conventional management strategies for comorbid shoulder spasticity and shoulder joint contractures typically combine physical therapy, pharmacological treatments, and focal interventions such as botulinum toxin injections or phenolization. Botulinum toxin remains the cornerstone of focal spasticity management. However, it is not indicated for the treatment of an apparent or actual fixed contracture and may have limited applicability in severe spastic shoulder deformities. Regulatory and off-label considerations constrain its use in key shoulder muscles: the pectoralis minor remains off-label for botulinum toxin injection across all jurisdictions, and the pectoralis major is on-label for certain toxin formulations in select countries only. In clinical practice, many patients discontinue botulinum toxin therapy due to insufficient or diminishing efficacy, and maximum recommended doses are often reached before all clinically relevant muscle groups can be adequately treated [7]. These limitations highlight the imperative for multimodal, mechanism-based treatment strategies that can be applied dynamically as part of comprehensive spasticity management. When these approaches fail, surgical procedures, including neurotomy or surgical tendon lengthening, may be considered. In such cases, accessible structures like the pectoral tendon are often targeted for percutaneous release [8,9]. However, pursuing more effective and minimally invasive treatment options remains a clinical need.

In recent years, percutaneous cryoneurolysis and percutaneous needle tenotomy have emerged independently as innovative interventions for spasticity and contracture [10,11]. Cryoneurolysis, which entails the controlled application of extreme cold to peripheral nerves, offers the possibility of offering novel procedures for the treatment of specific nerves in a spastic extremity without the need for open surgery [12]. Conversely, percutaneous needle tenotomy presents a minimally invasive technique for addressing contractures directly within the musculotendinous unit [13]. A recent case report successfully introduced the concept of combining these approaches to improve the range of motion in the adducted shoulder [14]. By analyzing a larger cohort, we aimed to determine the potential added value of the combination of percutaneous cryoneurolysis and percutaneous needle tenotomy to treat patients presenting with shoulder spasticity and concurrent pectoral tendon contracture.

The primary objective of this retrospective cohort study was to evaluate the combined effects and practical sequencing of cryoneurolysis and percutaneous pectoral tenotomy in patients with refractory shoulder spasticity and confirmed contracture on range of motion. The secondary objectives were to determine the combined effects of cryoneurolysis and percutaneous pectoral tenotomy on spasticity and to assess whether the addition of a pectoral tenotomy yielded substantially improved outcomes.

## 2. Results

### 2.1. Population Description

Participant demographic information by group is reported in Table 1. Fifteen patients (stroke: 11; MS: 2; CP: 2) presented with concurrent shoulder spasticity and pectoral tendon retraction, confirmed by Diagnostic Nerve Block (DNB), were enrolled in the study. One patient received treatment to both arms, which were considered separately in analysis. Four patients received both cryoneurolysis and tenotomy on the same day, and 11 patients received the treatments on separate days, all of whom received cryoneurolysis first and tenotomy second. For the patients having received treatments on separate days, the mean time between treatments was 410 days (sd = 526.5; min = 41; max = 2071).

### 2.2. Combined Effects of Cryoneurolysis and Tenotomy

The combined effect of cryoneurolysis and tenotomy on maximum passive range of motion was assessed with a repeated measures ANOVA for participants who received the treatments during two separate sessions. They demonstrated a significant increase over time for X(V1) for shoulder flexion (F = 14.43, *p* < 0.0001, ηp2 = 0.55, 95% CI = [0.38, 0.74]), and abduction (F = 11.22, *p* < 0.0001, ηp2 = 0.48, 95% CI = [0.33, 0.68], but not for external rotation (F = 1.03, *p* = 0.40, ηp2 = 0.11, 95% CI = [0.02, 0.52]). These findings are reported in Table 2.

Bonferroni-corrected Wilcoxon signed rank tests demonstrated a significant decrease in spasticity severity for shoulder flexion (W = 0.0, adj. *p* < 0.01, rbb = −0.86, 95% CI = [−1.00, −0.64]), abduction (W = 1.50, adj. *p* < 0.01, rbb = −0.77, 95% CI = [−1.00, −0.39]), and for external rotation (W = 0.0, adj. *p* < 0.05, rbb = −0.82, 95% CI = [−1.00, −0.63]) after both treatments compared to baseline. These findings are reported in Table 3.

To include participants who received both treatments on the same day and consequently did not record an observation for intermediate time points, paired *t*-tests were conducted including all participants regardless of timing, demonstrating a significant increase compared to baseline after all treatments for shoulder flexion (T = 5.95, *p* < 0.001, Cohen’s D = 1.44, 95% CI = [0.99, 2.45]), and abduction (T = 4.49, *p* < 0.005, Cohen’s D = 1.09, 95% CI = [0.63, 1.86]), but not external rotation (T = 1.2, *p* > 0.1, Cohen’s D = 0.32, 95% CI = [−0.15, 1.10]). These findings are reported in Table 4.

### 2.3. Effect of Cryoneurolysis Alone

Participants who received both cryoneurolysis and tenotomy on the same day were excluded from analysis as they did not include an observation for intermediate time points, which meant that the effect of cryoneurolysis alone could not be determined. For the participants who received treatments on separate days, X(V1) improved significantly for shoulder flexion (T = 5.13, adj. *p* < 0.001, Cohen’s D = 1.42, 95% CI = [0.75, 3.63]), and abduction (T = 4.49, adj. *p* < 0.005, Cohen’s D = 1.25, 95% CI = [0.76, 2.29]), but not external rotation (T = 1.23, adj. *p* > 0.1, Cohen’s D = 0.35, 95% CI = [−0.21, 0.89]) as per Bonferroni corrected paired *t*-tests (Table 3).

The same participants also exhibited significant decreases in spasticity severity for shoulder flexion (W = 0.00, adj. *p* < 0.05, rbb = −0.64, 95% CI = [−0.91, −0.36]), but not for abduction (W = 1.00, adj. *p* = 0.05, rbb = −0.55, 95% CI = [−0.91, −0.09]), or external rotation (W = 0.00, adj. *p* = 0.05, rbb = −0.88 [−1.00, −0.63]) as per Bonferroni-corrected Wilcoxon signed rank tests. However, both abduction and external rotation trended towards significance (Table 4).

### 2.4. Effect of Tenotomy Performed After Cryoneurolysis on a Separate Day

Due to the study design, the isolated effects of tenotomy could not be determined, insofar as cryoneurolysis was always performed first. Patients who received treatments on separate days underwent an assessment after cryoneurolysis, and another assessment immediately before tenotomy. These observations were compared with a Bonferroni-corrected paired T-test for X(V1) and Wilcoxon signed rank test for spasticity severity. There was no significant difference in spasticity severity for shoulder flexion, abduction, or external rotation. For X(V1) there was a significant difference for shoulder abduction (T = −3.50, adj. *p* < 0.05, Cohen’s D = −0.97, 95% CI = [−1.64, −0.61]) but not for flexion or external rotation (Table 4).

The effects of tenotomy were also compared with Bonferroni-corrected paired *t*-tests and Wilcoxon signed rank tests respectively. There were no additional improvements in X(V1) for shoulder flexion (T = 2.65, adj. *p* = 0.06, Cohen’s D = 0.74, 95% CI = [0.20, 1.58]), abduction (T = 0.59, adj. *p* > 0.1, Cohen’s D = 0.16, 95% CI = [−0.42, 1.58]), or external rotation (T = 0.52, adj. *p* > 0.1, Cohen’s D = 0.15, 95% CI = [−0.45, 0.87]) after tenotomy, though shoulder flexion range trended towards significance (Table 4).

There was also no additional decrease in spasticity severity after tenotomy after cryoneurolysis for shoulder flexion (W = 2.50, adj. *p* > 0.1, rbb = −0.50 [−0.90, 0.00]), abduction (W = 3.00, adj. *p* > 0.1, rbb = −0.50, 95% CI = [−0.90, 0.00]), or external rotation (W = 10.50, adj. *p* > 0.1, rbb = −0.11 [−0.67, 0.44]) (Table 3).

### 2.5. Adverse Events

There were no adverse events reported by any patients during or after cryoneurolysis. One patient presented with a hematoma after tenotomy due to unrecognized anticoagulant treatment. There were no consequences for the patient and the hematoma spontaneously resolved after 10 days. Pain during the procedure was commonly reported.

## 3. Discussion

This proof-of-concept cohort suggests that a sequential diagnostic and therapeutic strategy involving DNB, percutaneous cryoneurolysis and percutaneous tenotomy is both feasible and clinically beneficial for patients with combined spasticity and pectoral tendon contracture. Post hoc pairwise testing revealed that spasticity severity and range of motion changes occurred after cryoneurolysis alone. The purpose of tenotomy is to lengthen the myotendinous complex so as to allow for further range of motion at end range. As a result, no further reduction or improvement in the external range of motion was expected following the procedure, which was performed with the elbow flexed at 90°. However, even though, after multiple comparison correction, the change in range of motion for shoulder flexion after tenotomy was not statistically significant, the 95% confidence interval of the effect size highlights the likelihood of a positive effect; further investigation is warranted.

So as to deliver optimal care, clinicians who utilize botulinum toxin for spasticity management must be prepared to provide (or refer for) treatment other than chemodenervation. A large-scale multinational physician survey found that clinicians estimated that approximately 24.6% of patients with post-stroke spasticity would benefit from higher botulinum toxin doses than those permitted under current regulatory constraints, which circumscribe their ability to treat all clinically relevant muscle groups [7]. These real-world limitations underscore a need for multimodal spasticity management strategies extending beyond toxin-based interventions alone. As emphasized by Li et al. [15] comprehensive spasticity care requires familiarity with (and access to) non-toxin modalities in view of addressing neural, structural, and biomechanical contributors to impairment.

Tenotomy is more invasive than cryoneurolysis and is not required for all patients. Cryoneurolysis results in reversible nerve alteration, whereas tenotomy is characterized by a definitive approach to damage to the musculotendinous unit. Cryoneurolysis is therefore performed before tenotomy so as to manage spasticity before addressing tendon contracture. Several case studies have reported the benefits of cryoneurolysis on spasticity, lasting from months to years [16,17,18,19]. Furthermore, a recent cohort study demonstrated significant improvement in spasticity severity and range of motion in several movements of the upper extremity lasting one year after treatment [12]. In the current cohort, cryoneurolysis alone was sufficient to significantly decrease spasticity severity for shoulder flexion, abduction, and external rotation, and also to increase PROM for flexion and abduction. These findings suggest that a contracture does not preclude patients from achieving marked improvement from cryoneurolysis. Patients showing improved PROM from DNB, even those with contracture, may benefit from cryoneurolysis. Tenotomy was still considered for the patients in the current study because, although range of motion and spasticity improved, it had not improved as much as expected, likely due to the tendon contracture.

While spasticity and PROM improved after cryoneurolysis, in the 11 separate day cases, spasticity did not further improve after tenotomy. As stated above, however, the confidence interval around the effect might reflect minor range of motion improvement after tenotomy for either flexion and abduction. There are several possible explanations for this. First, as evidenced by the change in passive range of motion for shoulder flexion trending towards significance, and given the smaller effect sizes of changes observed after tenotomy, the sample size was plausibly too small to detect the difference; in any event, this cohort study should be used to power future investigations. A second possibility is related to the evident duration of effects from cryoneurolysis. As shown in the results, there was a significant decrease in range of motion for shoulder abduction after cryoneurolysis before tenotomy only over the mean 410 days between treatments. This implies that the treatment effect of cryoneurolysis was weaning for shoulder abduction over that time. As such, additional effects of the tenotomy may have been hidden by the already improved “new baseline”. Based on these preliminary and exploratory findings, randomized trials are needed to fully understand the effects of the individual treatments in isolation.

The decision to provide both treatments on the same day or separate days depended on several clinical factors. For instance, since tenotomy can be painful, high pain during cryoneurolysis may preclude patients from receiving tenotomy on the same day. That said, additional patient burden may not outweigh the potential benefit of PROM improvement. If additional treatment is required but pain was high, several periprocedural pain mitigation strategies have been tested. For example, distraction via virtual reality has been achieved during toxin botulinum injections, and could be considered for cryoneurolysis or tenotomy as well [20]. For most patients presenting with spasticity and tendon contracture, we recommend tenotomy soon after cryoneurolysis so as to hasten the final effects. Minimizing the time lapse between cryoneurolysis and tenotomy may provide greater benefits early in the patient pathway.

While pain was commonly reported during cryoneurolysis, there were no side effects or adverse events reported after the procedure. One patient experienced a hematoma after their tenotomy and recovered fully without any treatment after 10 days. For tenotomy, we recommend carefully screening patients to exclude those on anticoagulant medications and taking extra precautions when treating them. The absence of serious adverse events demonstrates the safety of the cryoneurolysis procedure for motor nerves [21].

### Limitations

The retrospective cohort design of the study, small sample size, heterogeneous timing of interventions, and lack of a control group constitute the major limitations of our study. Furthermore, the sequential treatment of cryoneurolysis followed by tenotomy precluded the isolation of tenotomy’s independent effects. Consequently, our findings reflect our routine clinical practice and the added value of tenotomy following cryoneurolysis. Although the improvements in MAS and PROM were clinically useful, the absence of functional measures limits the ability to determine the real-life impact of the intervention. While this preliminary work provides encouraging signals, they are not sufficient to draw definitive conclusion regarding meaningful functional benefits for patients. Future studies should include standardized functional scales (DASH, WHODAS, GAS) as well as measurements of pain and caregiver burden, the objective being to better capture the global relevance of the intervention in daily activities and participation. Future investigations can use this study to power randomized controlled trials designed to more closely explore the effects of each treatment individually, and in conjunction with each other.

## 4. Conclusions

Combined cryoneurolysis and percutaneous tenotomy was associated with significant improvements in spasticity severity and passive shoulder range of motion in patients with concurrent spasticity and contracture. While cryoneurolysis alone produced most of the measurable improvement, percutaneous tenotomy might provide additional end-range gains in selected patients, particularly when fixed contracture persists despite spasticity reduction. We acknowledge that our study trended towards a positive outcome, but this was not statistically significant for the 11 patients treated. Given the small sample size and heterogeneity in treatment sequencing, definitive conclusions about the individual contributions of each procedure cannot be drawn. Furthermore, it is important to note that the observed improvements in passive range of motion and spasticity do not necessarily translate into functional gains or enhanced quality of life for the patients. These preliminary and exploratory findings nonetheless support further investigation, including randomized controlled, function-oriented studies that incorporate assessment of patients’ quality of life, to better define the respective and combined roles of these interventions and to assess their applicability at other joints affected by contracture.

## 5. Materials and Methods

### 5.1. Design

This retrospective, single-center cohort proof-of-concept study was conducted to analyze the effects of combining pectoral nerve cryoneurolysis and percutaneous tenotomy in adult patients with refractory shoulder spasticity and pectoral tendon contracture. The study complied with the Declaration of Helsinki and was approved by the local research ethics boards (H23-00533). Data was collected between September 2023 and May 2024. Data are presented in accordance with the STROBE reporting guidelines for observational studies [22].

### 5.2. Participant Selection

The retrospective cohort included patients with shoulder spasticity who were referred to a single multidisciplinary spasticity clinic. To be eligible, patients (a) had refractory spasticity and were treated with cryoneurolysis as part and parcel of their routine care; (b) underwent an additional percutaneous pectoral tenotomy; and (c) were 19 years of age or older. Exclusion criteria included any prior history of surgery in the targeted area (such as tendon release or nerve surgery) and any history of botulinum toxin injection within the preceding four months. The exclusion of BoNT injections within four months was required to avoid residual pharmacological effects interfering with cryoneurolysis outcomes. While all included patients had previously received BoNT injections, they were classified as refractory due to insufficient clinical benefits.

Patients were selected to undergo cryoneurolysis after demonstrating a positive response to an ultrasound- and electrical-stimulation-guided Diagnostic Nerve Block (DNB) with 1–2 mL of lidocaine. A positive response was considered when the DNB demonstrated temporary improvement in spasticity and Passive Range Of Motion (PROM), previously demonstrated to predict the result of spasticity treatment with botulinum toxins and neurolytic interventions [23,24,25]. After cryoneurolysis demonstrated an improved, but not quite satisfactory range of motion increase based on clinical decision and patient satisfaction, reassessment was performed through a clinical evaluation in which manual palpation of the anterior axillary region revealed a prominent pectoralis major tendon, indicating musculotendinous retraction. Percutaneous tenotomy was proposed and performed to the patient so as to treat the residual musculotendinous retraction at the same day or on separate days from cryoneurolysis. Clinical assessments were performed before and after cryoneurolysis with or without combined tenotomy, and at follow-up visit. For a small subset of patients, tenotomy was performed on the same day as cryoneurolysis. In these cases, only pre-treatment and post-treatment assessments were completed.

### 5.3. Diagnostic Process and Treatment Algorithm

1. Initial assessment: Spasticity severity, PROM limitation, pain evaluation, and tendon palpation.
2. DNB (1–2 mL lidocaine): Used to predict responsiveness of pectoral nerve–mediated spasticity to cryoneurolysis.
3. Cryoneurolysis: Performed in patients with positive DNB.
4. Re-evaluation of PROM: If a mechanical “hard stop” persisted without spastic catch, pectoral tendon contracture was diagnosed.
5. Tenotomy: Performed either immediately or at a later session depending on patient tolerance.

### 5.4. Cryoneurolysis for Spasticity

Percutaneous cryoneurolysis was performed on the motor nerve branches to the lateral pectoral nerve to pectoralis major and medial pectoral nerve to the pectoralis minor with the Iovera device (Pacira, Parsippany, NJ, USA). Certain patients also received cryoneurolysis to additional motor and mixed sensorimotor nerves in the upper extremity as part of their treatment protocol (Table 5, please refer to Hashemi et al. [12] for further details). Surface anatomy was used to place the ultrasound probe, after which the targeted nerve was selected. A small amount of lidocaine was used to anesthetize the skin, and then a 16-gauge angiocatheter was inserted to protect the skin and assist in guiding the cryoprobe. E-stimulation was used to confirm the targeted location. A 106-s freezing and thawing cycle was performed for 1–3 lesions per target. Any side effects or adverse events during or after cryoneurolysis were recorded. Refer to the supplementary video S1 for a demonstration of shoulder cryoneurolysis.

### 5.5. Tenotomy for Pectoral Tendon Contracture

After cryoneurolysis, if a further increased range of motion of the shoulder was desired, a clinical examination was performed. If the passive range of motion showed that there was no longer a spastic catch through the range but rather a hard endpoint, manual palpation was used to determine the presence of a palpable pectoral tendon causing the limited range. If a spastic catch was detected, a second DNB was performed on additional muscles, such as the latissimus dorsi, teres major or subscapularis muscles, the objective being to determine whether existing spasticity called for additional treatment [26]. The percutaneous tenotomy procedure was provided so as to ensure pectoralis major tendon lengthening for patients, without the spastic catch. Anatomical location with palpation and ultrasound examination were carried out to confirm the tendon target and avoid blood vessels. The region was prepared with 2% chlorhexidine. Three ccs of 1% lidocaine were infiltrated into the skin and the targeted section of the tendon with ultrasound guidance. A 16-gauge needle was inserted into the distal end of the pectoralis major tendon. A partial section was carefully performed perpendicularly, scraping over the tendon. Any side effects or adverse events after tenotomy were recorded. Refer to the supplementary video S1 for a demonstration of percutaneous needle tenotomy.

### 5.6. Outcomes

Spasticity severity was assessed by (i) the degree of shoulder spasticity using the Modified Ashworth Scale (MAS) and (ii) the Passive Range of Motion (PROM) using manual goniometer collected by a physiatrist and a research assistant. Assessments were performed in shoulder flexion, shoulder abduction and external rotation.

### 5.7. Statistical Analysis

Statistical analyses were conducted using Python, version 3.11.7, with the following libraries: numPy version 1.26.4 and pandas version 2.1.4 for data manipulation; sciPy version 1.11.4 for modeling and post hoc statistical tests. For continuous outcomes (range of motion), a repeated measures analysis of variance (rmANOVA) was conducted to assess change over time after both treatments. Partial eta squared (ηp2) with 95% confidence intervals (95% CI) were reported. Paired *t*-tests were conducted to assess change after individual treatments and to include participants who received both treatments on the same day (and consequently did not record observations between treatments) and Bonferroni adjusted for multiple comparisons. For ordinal outcomes (MAS; 1+ converted to 1.5 for analysis), Bonferroni corrected Wilcoxon Signed Rank Tests were used to assess overall change and change from individual treatments. Rank-biserial correlation (rrb) was computed to quantify effect size of each treatment and was reported with a 95% CI.

## Figures and Tables

**Table 1:** Summary of participant demographics.

Median Age (IQR)	65.6 years (53.68)
Sex	Female: 9/Male: 6
Diagnosis	Stroke: 9
	Multiple Sclerosis: 2
	Acquired Brain Injury: 2
	Cerebral Palsy: 2
	Corticobasal Syndrome: 1

IQR: Interquartile Range.

**Table 2:** Change over time in maximum passive shoulder range of motion (X(V1)) (flexion, abduction and external rotation) after cryoneurolysis and tenotomy performed on separate days as assessed with a repeated measures analysis of variance.

Movement	Flexion	Abduction	External Rotation
F statistic (*p*-value)	14.43 (<0.0001)	11.22 (<0.0001)	1.03 (0.40)
Partial Eta Squared [95% CI]	0.55 ** [0.38, 0.74]	0.48 ** [0.33, 0.68]	0.11 * [0.02, 0.52]

* Medium effect size ** Large effect size.

**Table 3:** Change in spasticity severity (MAS) in shoulder flexion, abduction and external rotation between baseline and after all treatments. Pairwise comparison included baseline, post-cryoneurolysis, pre-tenotomy, and post-tenotomy regardless of tenotomy timing relative to cryoneurolysis as assessed with Bonferroni corrected (n_tests_ = 4) Wilcoxon signed rank tests.

Comparison	Baseline After All Treatments	Baseline After Cryoneurolysis	After Cryoneurolysis Before Tenotomy	After Cryoneurolysis After Tenotomy
W-statistic (adj. *p*-value)				
Flexion	0.00 (<0.01)	0.00 (<0.05)	7.00 (>0.1)	2.50 (>0.1)
Abduction	1.50 (<0.01)	1.00 (0.05)	6.5 (>0.1)	3.00 (>0.1)
External rotation	0.00 (<0.05)	0.00 (0.05)	4.00 (>0.1)	10.50 (>0.1)
rbb [95% CI]				
Flexion	−0.86 [−1.00, −0.64]	−0.64 [−0.91, −0.36]	0.00 [−0.46, 0.46]	−0.50 [−0.90, 0.00]
Abduction	−0.77 [−1.00, −0.39]	−0.55 [−0.91, −0.09]	0.00 [−0.46, 0.46]	−0.50 [−0.90, 0.00]
External rotation	−0.82 [−1.00, −0.63]	−0.88 [−1.00, −0.63]	0.14 [−0.43, 0.71]	−0.11 [−0.67 0.44]
IQR_D_				
Flexion	0.88	1.5	0.75	1
Abduction	1.00	2	0.75	1
External rotation	1.00	1	1.25	1

Adj.: adjusted; IQR: Interquartile Range; rbb = Rank-Biserial Correlation; 95% CI = 95% Confidence Interval.

**Table 4:** Change in maximum passive range of motion (X(V1)) in shoulder flexion, abduction and external rotation between baseline and after all treatments. Pairwise comparison included baseline, post-cryoneurolysis, pre-tenotomy, and post-tenotomy regardless of tenotomy timing relative to cryoneurolysis as assessed with Bonferroni corrected (n_tests_ = 4) paired *t*-tests.

Comparison	Baseline After All Treatments	Baseline After Cryoneurolysis	After Cryoneurolysis Before Tenotomy	After Cryoneurolysis After Tenotomy
T-statistic (adj. *p*-value)				
Flexion	5.95 (<0.001)	5.13 (<0.001)	−1.24 (>0.1)	2.65 (0.06)
Abduction	4.49 (<0.005)	4.49 (<0.005)	−3.50 (<0.05)	0.59 (>0.1)
External rotation	1.2 (>0.1)	1.23 (>0.1)	−0.78 (>0.1)	0.52 (>0.1)
Cohen’s D [95% CI]				
Flexion	1.44 [0.99, 2.45]	1.42 [0.75, 3.63]	−0.34 [−1.10, 0.20]	0.74 [0.20, 1.58]
Abduction	1.09 [0.63, 1.86]	1.25 [0.76, 2.29]	−0.97 [−1.64, −0.61]	0.16 [−0.42, 0.82]
External rotation	0.32 [−0.15, 1.10]	0.35 [−0.21, 0.89]	−0.26 [−0.77, 0.64]	0.15 [−0.45, 0.87]
Standard Deviation				
Flexion	19.37	11.87	13.46	13.52
Abduction	21.07	15.92	10.70	9.69
External rotation	25.62	21.65	21.42	20.44

Adj.: adjusted.

**Table 5:** Summary of nerves treated with cryoneurolysis for individual patients.

Participant ID	Lateral Pectoral Nerve	Medial Pectoral Nerve	Supra- Scapular Nerve	Sub- Scapular Nerve	Thoraco- Dorsal Nerve	Musculo- Cutaneous Nerve	Axillary Nerve	Median Nerve	Ulnar Nerve	Radial Nerve
1	X	X	X							
2	X	X								
3	X	X				X				
4	X	X				X		X		
5	X	X		X	X					
6	X	X	X	X		X	X	X		
7	X	X			X					
8	X	X	X	X			X			X
9	X			X	X	X	X	X	X	
10	X								X	
11	X	X	X			X		X	X	X
12	X	X		X		X		X	X	
13	X	X								
14	X	X	X						X	X
15	X	X				X		X		X
16	X	X	X					X		

## Data Availability

The data presented in this study are available on request from the corresponding author in accordance with institutional rules.

## Supplementary Materials

The following are available online at https://www.mdpi.com/article/10.3390/toxins18030137/s1, Video S1: An Innovative Approach to Treating Spastic Shoulder Contractures.

## Abbreviations

The following abbreviations are used in this manuscript:

CP	Cerebral Palsy
DNB	Diagnostic Nerve Block
IQR	Interquartile Range
MAS	Modified Ashworth Scale
MS	Multiple Sclerosis
PROM	Passive Range of Motion
W	Wilcoxon Test Statistic
T	T statistic
rbb	Rank-Biserial Correlation
95% CI	95% Confidence Interval
